# Phosphorus Limitation Enhances Diazotroph Zinc Quotas

**DOI:** 10.3389/fmicb.2022.853519

**Published:** 2022-04-21

**Authors:** Xuechao Wang, Thomas J. Browning, Eric P. Achterberg, Martha Gledhill

**Affiliations:** GEOMAR Helmholtz Centre for Ocean Research Kiel, Kiel, Germany

**Keywords:** *Trichodesmium*, dissolved organic phosphorus, alkaline phosphatase, zinc, iron, nitrogen fixation, diazotroph

## Abstract

*Trichodesmium* spp. is a colonial diazotrophic cyanobacterium found in the oligotrophic (sub)tropical oceans, where dissolved inorganic phosphorus (DIP) can be depleted. To cope with low P concentrations, P can be scavenged from the dissolved organic P (DOP) pool. This requires the deployment of multiple enzymes activated by trace metals, potentially enhancing metal requirements under stronger P limitations. To test this, we grew *Trichodesmium* under trace-metal-controlled conditions, where P was supplied as either DIP or DOP (methylphosphonic acid). Mean steady-state biomass under the DOP treatment was only 40% of that grown under equivalent DIP supply, carbon normalized alkaline phosphorus activity was elevated 4-fold, and the zinc (Zn)–carbon ratio was elevated 3.5-fold. Our finding matches the known, dominant Zn requirement across a diversity of enzymes involved in P stress responses and supports an important interaction in the oceanic cycles of these two nutrients.

## Introduction

Bioavailable nitrogen (N) is the limiting factor for phytoplankton growth throughout the majority of the (sub)tropical oceans (Moore et al., 2013). Diazotrophs, N_2_ fixing microbes, supply new bioavailable N to such environments, thereby increasing the productivity of the whole community (Mahaffey, 2005). In regions where N_2_ fixation is enhanced, dissolved inorganic phosphorus (DIP) is typically strongly drawn down (Martiny et al., 2019), often becoming growth-limiting for diazotrophs and co-limiting for the rest of the phytoplankton community (Moore et al., 2013).

In addition to DIP, P is also available as dissolved organic P (DOP), which accounts for up to 70–90% of the total dissolved P in surface waters of low DIP regions (Karl and Björkman, 2002). DOP utilization is mediated by enzymes that liberate DIP (Dyhrman et al., 2006); these include phosphoester hydrolyzing enzymes [e.g., alkaline phosphatase (AP), phosphodiesterase, and 5’ nucleotidase] and phosphonate catabolic enzymes (e.g., C-P lyase, substrate-specific enzymes including phosphonoacetate hydrolase and phosphonatase). Among these, several are metalloenzymes, requiring metal cofactors (Duhamel et al., 2021). Specifically, (i) AP enzymes require zinc (Zn) and magnesium (PhoA; Kim and Wyckoff, 1991), or calcium and iron (Fe) (PhoD/X; Yong et al., 2014; Rodriguez et al., 2014); (ii) phosphonoacetate hydrolases require Zn (McGrath et al., 2013); and (iii) C-P lyases contain Zn and Fe (Stosiek et al., 2020).

Alongside playing a role in regulating the rate of P acquisition from the DOP pool (Mahaffey et al., 2014; Browning et al., 2017), the trace element requirement for P releasing enzymes will also control the quotas of these elements in phytoplankton as a function of P limitation status. Altered trace element quotas should, in turn, feedback to the inventories and cycling of these elements in seawater (Duhamel et al., 2021). Clear evidence for this is, however, lacking. In this study, we use a new, trace-metal-clean culturing approach to examine the effects of different P sources on the growth of the diazotrophic cyanobacteria *Trichodesmium*. Our findings provide evidence for changes in micronutrient requirements, which are consistent with the diverse array of mechanisms used by marine microbes to alleviate P stress.

## Materials and Methods

### Cultures

To examine the effects of different P sources on the growth of the diazotrophic cyanobacteria *Trichodesmium* ISM101, we cultured *Trichodesmium* ISM101 with 5-μM DIP and 5-μM methylphosphonic acid (MPA) separately in triplicates using an exponentially fed-batch (EFB) culture system for 30 days. EFB cultures were carried out as described by Fischer et al. (2014) and Marki et al. (2020). For both treatments, *Trichodesmium* was cultured with 5-μM DIP before the start of our experiment. At the beginning of the experiment, the medium for one treatment was replaced with a medium with 5-μM MPA as the sole P source, the other treatment being kept at 5-μM DIP. Throughout the study, we followed trace metal clean protocols (Sunda and Huntsman, 2005). The cultures were maintained in EFB cultures at 25°C on a 12/12-h light/dark cycle at 140-μmol photons m^–2^ s^–1^ in YBCII media (Chen et al., 1996) with an amendment phosphorus concentration (5 μM) and a Fe concentration (40 nM). The YBCII medium was prepared with ultrapure water (Milli-Q, MQ; ≤ 18 MΩ cm^–1^; Millipore) and analytical reagent grade salts (Supplementary Table 1). YBCII medium was adjusted to pH 7.8–8.1 by addition of 0.01-M sodium hydroxide and filter sterilized with disposable rapid flow filter units (PES, 0.1 μm, Nalgene). A total of 10-L high-density polyethylene carboys (Nalgene) was used for the YBCII culture medium reservoir. The initial volume of the culture was 1,400 ml. Fresh medium was pumped into sterile polycarbonate culture bottles (2,000 ml, Nalgene), maintaining a constant dilution rate of 0.1 day^–1^ through narrow bore polypropylene tubing (inner diameter 0.51 mm, Ismaprene, Pharmed) using a peristaltic pump (IPC-N, Ismatec). The peristaltic pump was controlled by a programmable microcontroller (Raspberry Pi3), which automatically increased the flow rate each hour in relation to the real-time culture volume. The dilution rate used together with the starting volume allowed a single time point recovery of ∼500 ml of sample every 3 days. All sample collection and medium preparation was carried out in a laminar flow hood equipped with a high efficiency particulate air filter, located within a trace-metal-clean laboratory.

### Fast Repetition Rate Fluorescence

Fast repetition rate fluorometry of *Trichodesmium* ISM101 cultures was conducted throughout the whole culture period with a FASTOcean sensor equipped with a FASTAct laboratory system (Chelsea Technologies Group). Samples were dark acclimated for 20–30 min in the culture cabinet before measurements. Recovery of minimum fluorescence (*F*o) and maximum fluorescence (*F*m) allowed the determination of the potential photochemical efficiency of photosystem II (PSII) [*F*v/*F*m = (*F*m-*F*o)/*F*m].

### Alkaline Phosphatase Activities

Whole water alkaline phosphatase activities (APAs) rates were measured using the fluorometric substrate MUF-P (Sigma-Aldrich) following the protocol of Ammerman (1993). A total of 100-mM concentrated MUF-P and MUF (Sigma-Aldrich) stock solution was prepared by dissolving MUF-P and MUF into 2-methoxyethanol. Working stocks (100 μM) were made daily by diluting this concentrated stock with Milli-Q water. The assays were started by adding 100 μl of MUF-P to 5-ml samples in replicate 15-ml tubes to yield a final substrate concentration of 2 μM. A 150-μl subsample was transferred into a 96-well plate immediately from the mixed sample. Fifty microliters of filtered 50-mM borate buffer (pH 10.8) was added to the subsample in the well plate and mixed to a final pH > 10. APA was measured on a temperature-controlled (25°C) plate reader (FLX800TBI, BioTek) with Gen 5 software using an excitation wavelength of 365 nm and an emission wavelength of 455 nm. Fluorescence measurements were performed at *t* = 0, 1, and 2 h. APA (h^–1^) was calculated as the fluorescence of a 2-μM MUF divided by the initial (*t* = 0 to *t* = 2 h) slope of the fluorescence time course (fluorescence per hour). Regular Milli-Q blanks and paraformaldehyde-killed controls were conducted and generally yielded fluorescence values similar to *t* = 0 readings.

### Chlorophyll *a*

Twenty milliliters of the sample was filtered onto glass fiber filters (25 mm, 0.7 μm, Fisherbrand) and stored in a −80°C freezer until analysis. Samples were extracted for 24 h in 10-ml 90% acetone in a −20°C freezer in the dark. Samples were brought to room temperature in the dark before measurement on a calibrated Turner Designs trilogy fluorometer following the method of Welschmeyer (1994).

### Nutrients

Samples of dissolved inorganic nitrate + nitrite and P (15 ml) were filtered through glass fiber filters (25 mm, 0.7 μm, Fisherbrand) under low pressure (200 mpa). Samples were stored in polypropylene tubes (15 ml, Fisherbrand) at −20°C until analysis and then analyzed using a SEAL QuAAtro nutrient autoanalyzer system (SEAL Analytical). Samples of total dissolved P (TDP; 50 mL) were filtered through sterile PES syringe filters (0.2 μm, Fisherbrand). Samples were stored in polypropylene tubes (50 ml, Fisherbrand) at −20°C. Before analysis, TDP samples and blanks were digested under elevated pressure (1.5 bar) and temperature (120°C) for 30 min after addition of the oxidizing reagent Oxisolv (Merck) and then analyzed using a SEAL QuAAtro nutrient autoanalyzer system (SEAL Analytical). DOP concentration was subsequently calculated as DOP = TDP–DIP. After measuring 10 samples of YBCII medium with a fixed TDP concentration of 5 μM, we determined that the mean oxidation efficiency of TDP during the measurement was 86.35 ± 11.24%. Our DOP data were, therefore, corrected upward for this oxidation efficiency.

### Particulate Organic C/N

Particulate organic C/N concentrations were determined by filtering a 50-ml sample through pre-combusted (500°C, 12 h) glass fiber filters (25 mm, 0.7 μm, Fisherbrand) under low pressure (200 mpa). Filters were stored frozen at −20°C. Before analysis, filters were dried at 50°C for 12 h. After drying, filters were wrapped in tin boats (8 mm × 8 mm × 15 mm), put in a well plate, and stored in a desiccator. Samples and blanks were analyzed using an elemental analyzer (Eurovector EA3000 Elemental Analyzer) with Callidus version 5.1 software.

### Particulate Element Concentrations

Fifty milliliters of the sample was filtered onto acid-cleaned (10% HCl) PES membrane filters (25 mm, 0.8 μm, PALL). Filters were frozen and stored at −20°C until digestion. Cell digestions were carried out as described by Honey et al. (2013): filters were placed in acid-washed (20% HNO_3_) 15-ml PFA digestion vials with 2 ml of concentrated redistilled HNO_3_ (Savillex, QMX) and heated to 120°C for 24 h. The filter was removed from the vial and the HNO_3_ evaporated off (75°C, overnight). The residue was dissolved in 3 ml of 1% HNO_3_ and spiked with indium (4 μg L^–1^) as an internal standard. The samples were then centrifuged at 14,000 rpm (Centrifuge 5430R, Eppendorf) for 10 min, and the upper supernatant was taken for determination by inductively coupled plasma mass spectrometry (Element XR, Thermo), using prepared elemental standard curves for quantification.

## Results and Discussion

**FIGURE 1:**
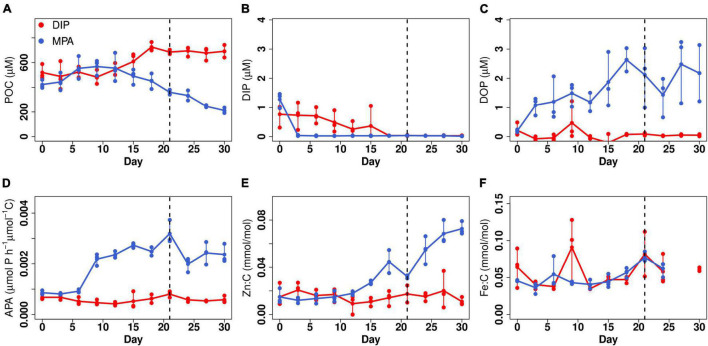
Responses of *Trichodesmium* ISM101 to DIP and MPA treatment. **(A)** Particulate organic carbon (POC). **(B)** Dissolved inorganic phosphorus (DIP). **(C)** Dissolved organic phosphorus (DOP). **(D)** POC-normalized APA. **(E)** Zn:C ratio. **(F)** Fe:C ratio. Points show triplicate measurements every 3 days; lines map change in mean value with time. Time to right side of dashed line is considered to be steady-state.

We tested for interactions between P limitation, DOP availability, and cellular elemental quotas in *Trichodesmium* ISM101 under two P supply treatments: (i) 5-μM DIP and (ii) 5-μM MPA, a phosphonate synthesized by marine microbes (Metcalf et al., 2012). Experiments were conducted at high volume (2 L) with triplicate replication under trace-metal-clean conditions using an EFB culture approach (Fischer et al., 2014; Marki et al., 2020), which allows for regular harvesting of sufficient biomass required for bulk phytoplankton elemental analysis. *Trichodesmium* biomass [particulate organic carbon (POC)] within the DIP treatment increased 1.4-fold over 18 days and showed no significant changes from day 12 (Quade *post hoc* test; *p* < 0.05; Figure 1A), after which we assumed that growth had reached an approximate steady-state matching the dilution rate of the culture (0.1 day^–1^), and biomass concentration was dictated by the concentration of the most deficient nutrient in the culture medium (Fischer et al., 2014). The biomass of the MPA treatment showed a more complex trend, with POC ultimately decreasing to 0.7 times the initial value within 21 days, after which no significant changes were observed, and a steady state was assumed (Quade *post hoc* test; *p* < 0.05; Figure 1A).

Concentrations of DIP for both treatments were fully depleted at a steady-state (0.03 ± 0.01 and 0.02 ± 0.01 μM; Figure 1B), implying that for both sets of cultures, the low P availability limited biomass. Nevertheless, three lines of evidence suggested that (i) the level of P stress in the MPA treatments was higher than that of the DIP treatment, and (ii) that this was due to a situation approaching P-Fe co-limitation in the DIP treatment. Firstly, APA was fourfold higher in the MPA treatment, implying stronger P stress in this treatment (Figure 1D; Mahaffey et al., 2014). Secondly, the apparent photochemical PSII efficiency (*F*_*v*_/*F*_*m*_) was significantly lower in the DIP treatment (Supplementary Figure 2B). *F*_*v*_/*F*_*m*_ has been previously demonstrated to remain high under steady-state P limitation (Kruskopf and Flynn, 2006), whereas under steady-state Fe limitation, large decreases are observed (Schrader et al., 2011). Thirdly, mean steady-state POC per filament in the DIP treatment was 45% lower than the MPA treatment (pairwise Wilcox test, *p* < 0.05), which we hypothesize was related to filament length, as *Trichodesmium* filaments have been shown to be significantly smaller under limitation by Fe in comparison with P (Tzubari et al., 2018). Fourth, the mean steady-state particulate Fe concentration in DIP treatment was 46 ± 16 nM, indicating that all of the supplied Fe in the medium (40 nM) was taken up by *Trichodesmium*. In turn, stronger P limitation in the MPA treatment could be ascribed to a lower accessibility of this P source (Dyhrman and Haley, 2006; Sosa et al., 2019), with dissolved MPA concentrations in this treatment remaining at around 40% of the supplied concentration (2.05 ± 0.93 μM; Figure 1C).

**FIGURE 2:**
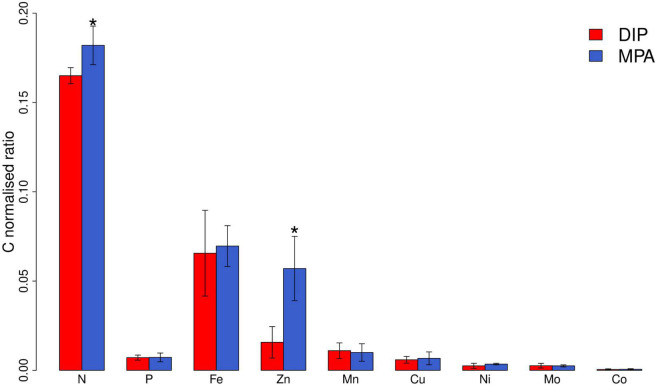
Elemental composition of *Trichodesmium* ISM101 at steady state (day ≥ 21) grown under supply of DIP and MPA. Bars show mean steady-state elemental ratios; error bars are standard deviation (*n* = 7–12). N and P are in units of mol:mol; all others are in units of mmol:mol. *Statistically significant differences between treatments (pairwise Wilcox test, *p* < 0.05). We note that mean steady-state particulate Fe:C ratios were similar to those found in literature for *Trichodesmium* (65.6 ± 24 μmol:mol in comparison with 69.5 ± 3.48 μmol:mol in Berman-Frank et al. (2001)).

Differences in elemental composition between the two treatments at steady state were minimal for all elements apart from Zn (Figure 2 and Supplementary Figure 1), which in contrast showed 3.5-fold elevated Zn:C in the MPA treatment (0.057 ± 0.018 mmol/mol C in comparison with 0.016 ± 0.009 mmol/mol C in the DIP treatment). This finding is consistent with a recent evaluation of trace element requirements for mechanisms to cope with P limitation (Duhamel et al., 2021): of all the trace elements assessed, Zn stands out as having the greatest demand, with elevated requirements in comparison with all other metals across a diversity of transferases, hydrolases, lyases, isomerases, ligases, and translocases (Duhamel et al., 2021). With our set of observations, we cannot decouple the specific cellular sinks for Zn that occurred within the MPA treatment. However, notable was that APA in MPA treatment increased from day 6 and stabilized from day 9 (Figure 1D), whereas the increase in Zn:C was later, from day 12, and did not reach a clear plateau over the experiment duration (30 days; Figure 1E). The latter observation suggests that the Zn enhancement was not simply due to the well-characterized enhanced production of AP enzymes under greater P stress (and specifically, Zn-requiring PhoA; Mahaffey et al., 2014) but rather to a diversity of strategies to cope with stronger P limitation (Wei et al., 2018; Duhamel et al., 2021). Furthermore, the lack of increase in Fe requirements under stronger P limitation (Figure 1F) is inconsistent with the expectation of more dominant roles of PhoX and PhoD over PhoA (Luo et al., 2011) but is consistent with (i) the Fe requirement for these enzymes being relatively minor in comparison to overall cellular Fe demands and (ii) the lower Fe—but dominant Zn—requirement in a variety of other, non-AP, enzymes produced in response to low P (Duhamel et al., 2021).

Our observations of enhanced Zn quotas under stronger P limitation in culture are consistent with available field observations (Tovar-Sanchez et al., 2006; Twining et al., 2010). Tovar-Sanchez et al. (2006) found that Zn:P quotas of *Trichodesmium* in the low P eastern Subtropical North Atlantic were ∼8.6 mmol mol^–1^, very close to the mean steady-state Zn:P quota that we observed in the MPA treatment (8 ± 3.7 mmol mol^–1^). Additionally, Twining et al. (2010) found that the Zn quotas of *Synechococcus* in Sargasso Sea eddies with lower DIP supply were roughly 20 times higher than those with higher DIP supply. Together, our observations in culture and these findings in the field suggest a crucial level of interaction between the cycles of these two nutrients in the ocean. The impacts of such an interaction might be exacerbated in a future ocean, where increasing atmospheric N inputs could drive a shift toward more widespread and stronger P stress (Moore et al., 2013).

## Data Availability Statement

The original contributions presented in the study are publicly available. This data can be found here: doi: 10.5281/zenodo.5607662.

## Author Contributions

TB, MG, and XW designed the study. XW performed culture work, data collection, analysis, and wrote the manuscript. TB, EA, and MG contributed editorial comments and approved the final version of the manuscript. All authors contributed to the article and approved the submitted version.

## Conflict of Interest

The authors declare that the research was conducted in the absence of any commercial or financial relationships that could be construed as a potential conflict of interest.

## Publisher’s Note

All claims expressed in this article are solely those of the authors and do not necessarily represent those of their affiliated organizations, or those of the publisher, the editors and the reviewers. Any product that may be evaluated in this article, or claim that may be made by its manufacturer, is not guaranteed or endorsed by the publisher.
