# Supplementary material for: Phosphorus Limitation Enhances Diazotroph Zinc Quotas
Source: Front Microbiol. 2022 Apr 21;13:853519. doi: 10.3389/fmicb.2022.853519 (PMC9069106; doi:10.3389/fmicb.2022.853519)
Supplement: Supplementary file 1 [file Data_Sheet_1.docx]

Supplementary Material

# Supplementary Figures and Tables

## Supplementary Figures


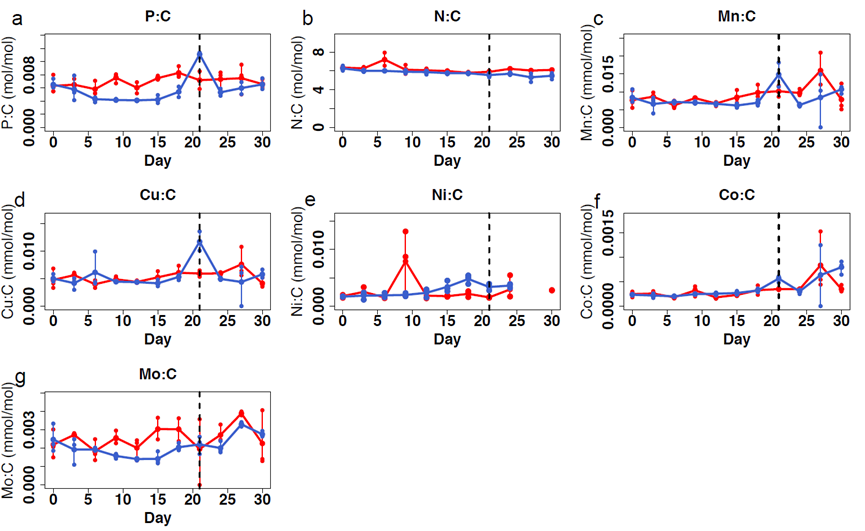


**Supplementary Figure 1.** Elemental ratios determined throughout the duration of the exponentially fed batch culture at steady state (day≥21) for *Trichodesmium* ISM101 growing with a constant dilution rate of 0.1 d^−1^ with 2 different P sources (DIP and MPA). Points show triplicate measurements every three days, lines map the change in the mean value with time, right side of the dashed line is considered to be the steady state.


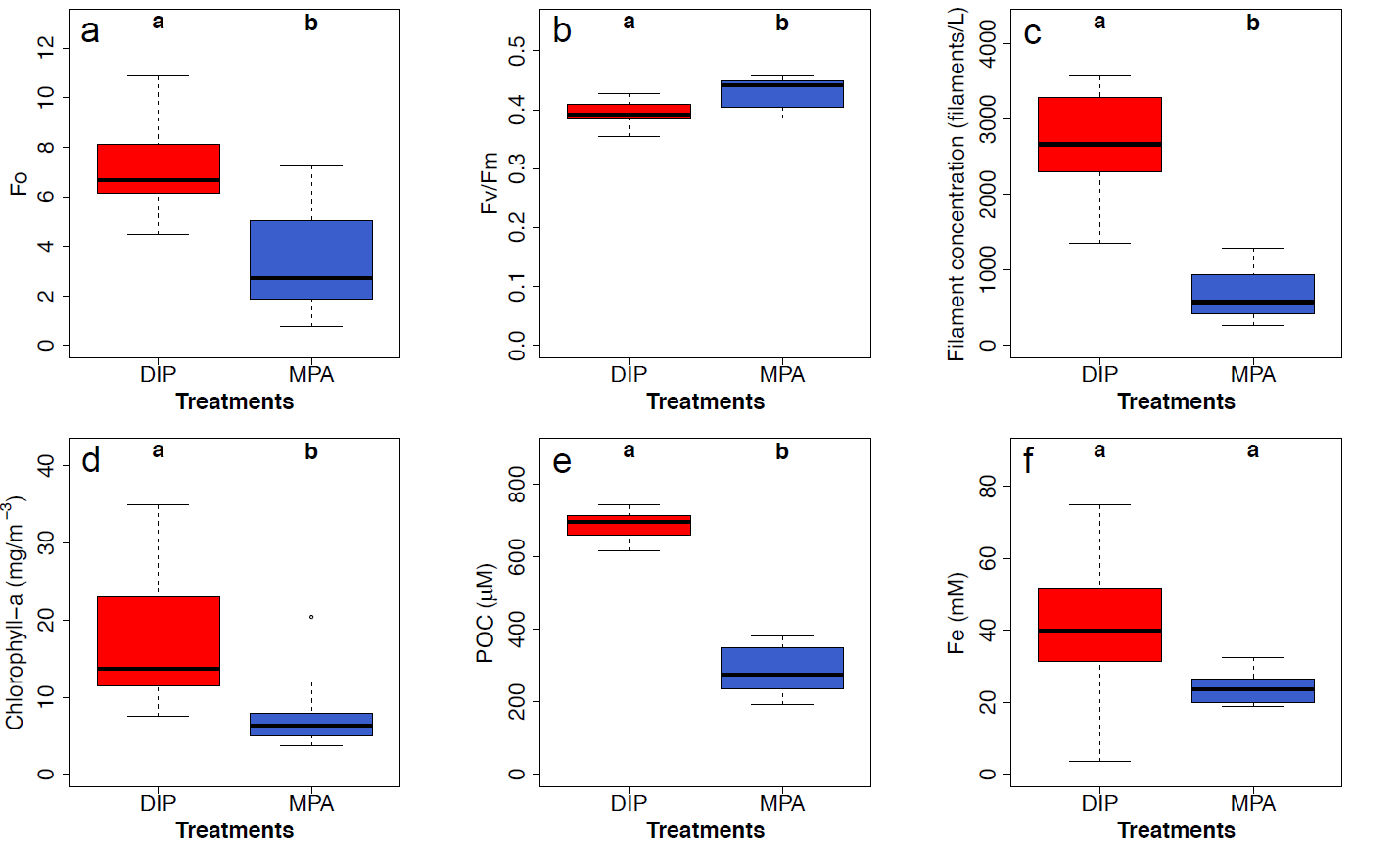
**Supplementary Figure 2.** Parameters determined at steady state (day≥21) for *Trichodesmium* ISM101 growing with a constant dilution rate of 0.1 d^−1^ with 2 different P sources (DIP and MPA). **a,** F_0_. **b,** Fv/Fm. **c,** Filament concentration. **d,** Chlorophyll a concentration. **e,** POC. **f.** Particulate Fe concentration. Letters indicate statistically significant differences between treatments (pairwise Wilcox test, p<0.05).

## Supplementary Figures

**Supplementary Table 1.** Modified YBCII media recipe used in our experiment

| Component | Concentration in media (mol L^-1^) |
| --- | --- |
| Major salt |  |
| NaCl | 0.42 |
| KCl | 0.01 |
| NaHCO_3_ | 0.0025 |
| H_3_BO_3_ | 0.00058 |
| KBr | 0.00097 |
| NaF | 0.00007 |
| MgSO_4_ | 0.025 |
| MgCl_2_ | 0.02 |
| CaCl_2_ | 0.01 |
| SrCl_2_ | 0.000065 |
| LiCl | 0.03 |
| Macronutrient |  |
| Na_2_EDTA·2H_2_O | 2.0×10^-6^ |
| CuSO_4_·5H_2_O | 8.0×10^-9^ |
| ZnSO_4_·7H_2_O | 2.0×10^-8^ |
| CoCl_2_·6H_2_O | 8.0×10^-9^ |
| MnCl_2_·4H_2_O | 1.8×10^-8^ |
| Na_2_MO_4_·2H_2_O | 1.0×10^-7^ |
| NiSO_4_·6H_2_O | 2.0×10^-8^ |
| Na_2_SeO_3_ | 1.0×10^-8^ |
| FeEDTA | 4.0×10^-8^ |
| Vitamin |  |
| Thiamine | 3.0×10^-7^ |
| Biotin | 2.0×10^-9^ |
| Cyanocobalamin | 3.7×10^-10^ |
